# Fast Object Motion Estimation Based on Dynamic Stixels

**DOI:** 10.3390/s16081182

**Published:** 2016-07-28

**Authors:** Néstor Morales, Antonio Morell, Jonay Toledo, Leopoldo Acosta

**Affiliations:** Departamento de Ingeniería Informática, Universidad de La Laguna, Avda. Astrofísico Francisco Sánchez, s/n, San Cristóbal de La Laguna 38271, Spain; amorell@isaatc.ull.es (A.M.); jonay@isaatc.ull.es (J.T.); leo@isaatc.ull.es (L.A.)

**Keywords:** stixels, object tracking, object clustering, 3D reconstruction, autonomous vehicles

## Abstract

The stixel world is a simplification of the world in which obstacles are represented as vertical instances, called stixels, standing on a surface assumed to be planar. In this paper, previous approaches for stixel tracking are extended using a two-level scheme. In the first level, stixels are tracked by matching them between frames using a bipartite graph in which edges represent a matching cost function. Then, stixels are clustered into sets representing objects in the environment. These objects are matched based on the number of stixels paired inside them. Furthermore, a faster, but less accurate approach is proposed in which only the second level is used. Several configurations of our method are compared to an existing state-of-the-art approach to show how our methodology outperforms it in several areas, including an improvement in the quality of the depth reconstruction.

## 1. Introduction

Considerable work has been carried out to improve the efficiency and performance of obstacle-detection methods applied to Advanced Driver Assistance Systems (ADAS). Many solutions are based on dense environment reconstruction using disparity maps. Although these methods are useful for a detailed understanding of the environment, the reconstruction is dense and relies heavily on computer resources. Minimizing the area of the image to be processed allows for a simpler and lighter reconstruction based on certain assumptions.

**Figure 1:**
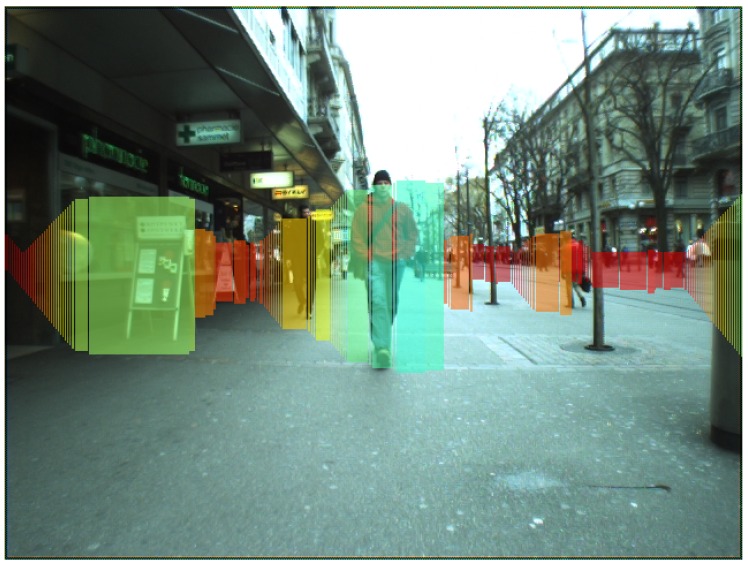
Stixels superimposed on their original image indicating detected obstacles. Colors encode the distance to the camera.

Given a 3D reconstruction of the world, typically from a stereo input, though it can be 3D LIDAR data, a depth camera or similar, the objective is to simplify the scene’s complexity by removing those parts of the environment with no information. The main objects in the scene are kept, but they are simplified. The model only focuses on the dominant objects in the scene, without a pixel-wise depth map, meaning the model can be estimated much faster than with traditional tracking methods. To this end, Badino et al. [1] proposed a representation of the world based on a set of rectangular sticks called stixels (from stick and pixel). Each stixel is defined by its 3D position relative to the camera and stands vertically on the ground, having a certain height, as shown in Figure 1. This compact, but flexible representation of the world can be used as the common basis for scene understanding tasks. The stixels can be generated without calculating a depth map by using techniques, such as V-disparity—or column-wise disparity—[2], which also offers substantial computational advantages. This fact is also the main reason why the original implementations from [1,3] are not generally used.

The main advantages of using such an approach are:

- Compact: significant reduction in data volume.
- Complete: information of interest is preserved.
- Stable: small changes in underlying data do not cause rapid changes in the representation.
- Robust: outliers have little or no impact on the resulting representation.

**Figure 2:**
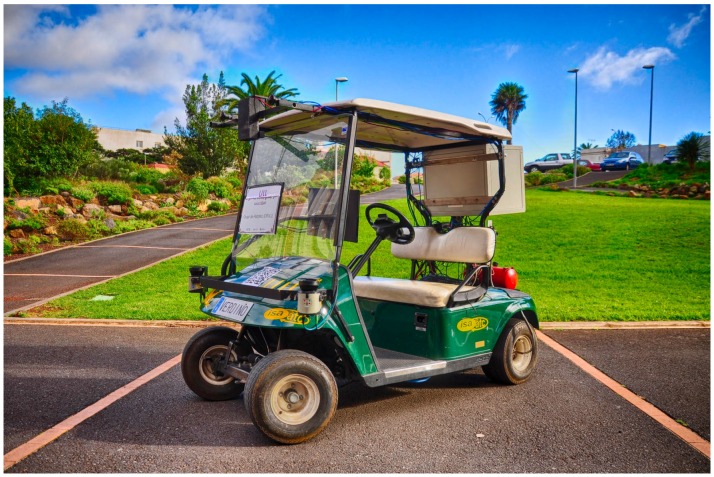
The algorithm is intended for the Verdino prototype, designed to travel in pedestrian environments.

This obstacle detection and tracking method has been developed as part of the obstacle detection subsystem of our autonomous vehicle (Verdino) [4] (shown in Figure 2). Verdino is an electric vehicle designed to transport people in different environments, including pedestrian streets or tourist resorts, without needing a driver. Therefore, its behavior must be mainly reactive, with safety as its top priority. It has been modified to be able to drive autonomously at a maximum speed of 6 m/s, operated by an onboard computer. To this end, the original steering, brakes and accelerator have been modified and various sensors mounted on it [5,6], including a stereo camera. A crucial task for safe navigation is environment reconstruction, obstacle detection and motion prediction, so that Verdino can safely avoid obstacles.

Tracking capabilities can be estimated by how stixels move between frames [7]. Stixels are valid for representing the area around a vehicle, and they provide enough detail for motion detection at a lower computational cost than optical flow where maximum object speed is limited. The contribution of this work can be summarized as:

- Good reconstruction quality in terms of computed depth. Free space computation without disparity maps has some drawbacks involving low depth accuracy. Object reconstruction and the detection scheme improve the correction of stixel depths and remove false obstacles.
- Better detection results and faster tracking than other methods, as in [7].
- Better robustness after changes between images (for example, when faced with a low frame rate).
- Stixel obstacle detection in crowded pedestrian areas provides reliability and speed at the same time.

In the next section, we discuss previous research on stixels. Section 3 describes the method pipeline. Section 4 presents a set of tests. Finally, conclusions are drawn in Section 5.

## 2. Previous Work

The problem of obstacle tracking has been well studied for its application in ADAS. In [8], a review of techniques applied to on-road systems, including vehicle detection, tracking and behavior understanding, is presented, making a special emphasis on vision-based algorithms. Many of these approaches use monocular vision for this task. An example is the work in [9], where lines painted on the road are detected by a single monocular camera, and an automatic steering control, speed assistance for the driver and localization of the vehicle are presented. In [10], the authors go one step further, trying to predict pedestrian behavior based on the Gaussian process, dynamical models and probabilistic hierarchical trajectory matching.

Stereo vision is also used to detect obstacles [11] using 3D information. Based on how much information they use, two subcategories can be found. First, there is a set of methods falling inside the category of 2.5D solutions. In this category, the complete information provided by 3D points is not used. Some of these methods use the 3D point as a feature, as in [12], in which dense variational optical flow estimation is combined with Kalman filtering for temporal smoothness and robustness. In [13], obstacles are represented as a rigid 3D point set, being tracked in terms of feature displacements and depth measurements. A very popular choice is the use of occupancy grids, like in [14,15]. About 3D solutions, they are usually based on complex grid maps that use complete 3D information. There are many ways of doing such a representation, i.e., with octree connected cubes [16] or voxel grids [17], used not only for stereo vision data [18]. This category includes sensor fusion approaches, like that in [19], where an obstacle tracking system for urban scenarios is made by a combination of odometry, LIDAR and computer vision, or in [20], where visible and FIR cameras are used to detect pedestrians.

Object tracking can be divided into online systems (for which tracking is done on a frame-by-frame basis), or offline systems (which take longer sequences into account), like in [21,22]. In the online systems, targets are usually followed using classic tracking approaches, like the Extended Kalman Filters (EKFs) [23], particle filters [24] or mean-shift tracking [25]. In [26], a simultaneously detection and trajectory estimation over a hypothesis test model extended with stereo depth and visual odometry is presented. Some solutions try to model the social behavior of the pedestrians in order to improve the obtained tracks, as happens in [27,28,29]. Other approaches use an intermediate solution between online and offline systems, like the Multi-Hypothesis Tracking (MHT) [30] or the Joint Probabilistic Data Association Filters (JPDAFs) [31].

Methods based on stixels [1,2,32,33] simplify the world defining only the 3D position relative to the camera and the height of the obstacle. Depending on how stixels are computed, two main trends emerge. In [3,34,35,36], free space is based on disparity maps, which use a probabilistic scheme to reduce the number of parameters. The number of objects captured along every column is assumed to be small. Flying objects are penalized, and elevated objects have higher depths than lower ones. The work in [35] improves on [34] by using three different stereo confidences. In [3], a free space scheme that is able to reduce computational costs with a Kalman filter for tracking and clustering stixels is presented. Finally, in [36] the probabilities of a collision in a roundabout are computed.

The other research line is based on free space computation without disparity maps. In [2,7,33,37], a very high frame rate is achieved using a Sum of Absolute Differences (SAD) cube, with a cost associated with each row, column and disparity combination. This cube is used to compute the v-disparity, yielding a ground plane model. Stixels are computed using the points at the boundary with the ground (obtained with Dynamic Programming (DP)), including the height limitations of expected obstacles and left-to-right occlusion constraints.

## 3. Method

**Figure 3:**
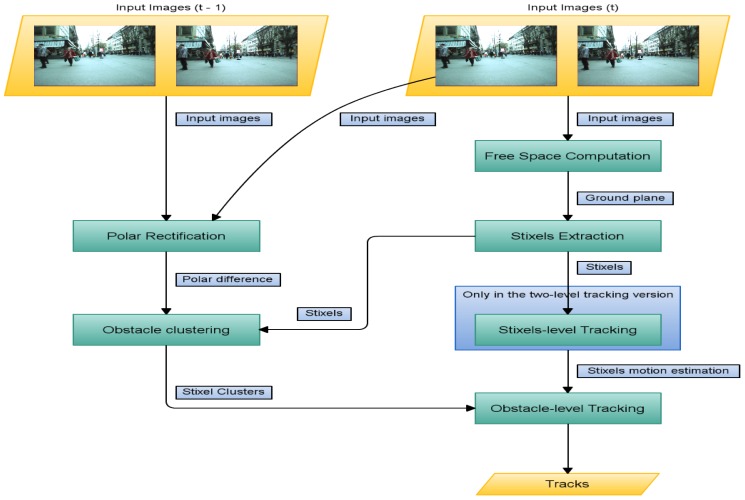
Graphical description of the method described in this paper.

The method described in Figure 3 consists of the following steps:

1. Free space is computed from a stereo pair in order to estimate the ground plane.
2. Stixels are obtained and placed on the ground based on their depth and position.
3. At the first level, the stixels are tracked as per [7]. The set of stixels in the current frame is compared and matched to the previous one.
4. Stixels are clustered based on their projected position in 3D.
5. Using these clusters and the tracked stixels, tracking is performed at the stixel level. Obstacles in the scene and their velocities are calculated, and their positions in previous frames are recorded to estimate their future motion.
6. In the second level, tracking is performed only at the object level. Each obstacle is compared to obstacles detected in previous frames, meaning that stixel-level tracking is no longer needed.

Open-loop tracking is used at both the obstacle and stixel levels in order to reduce the calculation time. To determine the next position, only elements in the current frame are considered and linked to the following frame. The steps are detailed in the sections that follow. In Section 4, the advantages and drawbacks of using either approach are detailed, and two-level-based tracking is described in the attached video (method pipeline). This pipeline is also valid for non-stixel based object tracking (see Section 3.3.2) if the first step of the algorithm is ignored.

### 3.1. Computing Stixels

Our stixel extraction method is similar to the one in [2], with the following assumptions:

- The algorithm’s input is a calibrated stereo image pair.
- A Lambertian surface is assumed.
- The ground is planar, at least locally.
- Objects are mainly vertical with a limited height.
- The stereo rig has negligible roll with respect to the ground plane.

#### 3.1.1. Computing the Free Space

The ground plane is estimated using data collected in the *v*-disparity domain [2]. Instead of computing and projecting a dense stereo depth map (much more computationally expensive), a function f(u,v)=D is obtained in which (u,v) is the pixel position and *D* is the disparity of this position. For each row, the disparity with the lowest cost is extracted, and the ground level is obtained by robustly fitting a line on the *v*-disparity image. For optimization purposes, only one row of each *N* (where *N* is the number of rows) is computed, and the ground plane is interpolated.

#### 3.1.2. Stixel Extraction

Stixel detection divides the image into multiple row bands $b_{i}$. Inside each band $b_{i}$ and for each column $u_{i}$, the pixel with the largest horizontal gradient is selected [33]. This reduces the computational cost while increasing accuracy and provides us with a set of possible locations in which the bottom coordinate of each stixel could be located. In the presence of a horizontal stripe that could confuse the algorithm (like, for example, in the presence of cobbles), errors will be bounded by the band height.

Having a set of potential row bands that could be used as the bottom coordinate of each stixel, the next step is to localize the optimal one. The likelihood of the presence of a stixel *q* at row band *b* is calculated based on the cost of the presence of a vertical object at that location; the probability of the supporting ground being present; and a smooth term to force the left-right occlusion restrictions, by promoting ground-object boundaries with few jumps. The ways in which these costs are computed are beyond the topics covered in this paper, but more information can be found in [2]. The minimum size of the stixel is set to 10 pixels. The results after this stage are shown in Figure 1, where stixels (in colored depth scale) are superimposed on the left image. More information is provided in Section 3.1 and Section 3.2 and in [2].

### 3.2. Tracking

Two different tracking approaches have been explored. The first one is based on two tracking levels. The first level tracks stixels independently (stixels in the current frame are matched with another, or none, in the previous frame by minimizing the cost function associated with matching two stixels). In [7], this is done using DP. In our implementation, a bipartite matching graph is used. In the second level, stixels are clustered into objects, which are matched based on the inner stixels previously tracked.

In the other approach, only the second level is performed. The tracking does not consider stixels included in objects. Stixels are only used in the clustering and reconstruction process. Section 3.2.1 applies only to the two-level approach, while Section 3.3 is common to both approaches.

For this stage, some assumptions were made:

- All stixels are assumed to be properly estimated.
- The maximum object speed is limited, so the search range between stixels is constrained. As there is just one stixel per column, matching is limited to a search in the *u* direction.
- Since two consecutive frames are relatively close, the same stixel at time *t* and t−1 should look similar, including its height. Section 4.3.2 shows that this restriction can be reduced depending on the tracking approach.

#### 3.2.1. Stixel-Level Tracking

The tracking objective is to match each stixel at column $q_{i}\left\{t\right\}$ with the corresponding stixel in the previous frame (t−1). This process can be thought of as a pair matching problem. A bipartite graph, in which the nodes are the stixels in frames *t* and t−1 and the edges are associated with a certain motion cost $c_{m}$, is used to match the stixels. This is represented by Equation (1).

(1) $$c_{m}\left(u_{i}\left\{t\right\},u_{j}\left\{t-1\right\}\right)=\begin{array}{c} \left\{\begin{array}{cc} f_{cost}\left(u_{i}\left\{t\right\},u_{j}\left\{t-1\right\}\right) & \;\text{matching}\\ \infty & \text{other} \end{array}\right. \end{array}$$

Here, a match is applicable if and only if the following restrictions are satisfied:

- $|X\left(u_{i}\left\{t\right\}\right)-X\left(u_{j}\left\{t-1\right\}\right)|<\tau_{max\_disp}$, where parameter $\tau_{max\_disp}$ indicates the maximum stixel displacement between frames; and X(u) is the position in 3D coordinates in the longitudinal axis *X*, which grows from left to right in 3D Cartesian coordinates. Axis *Y* is the vertical axis, which grows downwards, and the *Z* axis starts from the local coordinate system of the robot towards its front.
- $u_{i}\left\{t\right\}$ is not the first frame in which stixel $u_{i}\left\{t\right\}$ appears.
- Stixels $u_{i}\left\{t\right\}$ and $u_{j}\left\{t-1\right\}$ are not occluded.
- $f_{cost}\left(u_{i}\left\{t\right\},u_{j}\left\{t-1\right\}\right)<\tau_{max\_cost}$.

If a match is not found, the cost is infinite, and thus, the link is not included in the graph. The cost function is described in Equation (2).

(2) $$f_{cost}\left(u_{i}\left\{t\right\},u_{j}\left\{t-1\right\}\right)=\;c_{SAD}+c_{hist}+c_{height}$$

with:

(3) $$\begin{array}{cccc} & \alpha_{SAD} & \;\cdot \; & f_{SAD}\left(u_{i}\left\{t\right\},u_{j}\left\{t-1\right\}\right)\\ & \alpha_{hist} & \;\cdot \; & f_{hist}\left(u_{i}\left\{t\right\},u_{j}\left\{t-1\right\}\right)\\ & \alpha_{height} & \;\cdot \; & f_{height}\left(u_{i}\left\{t\right\},u_{j}\left\{t-1\right\}\right) \end{array}$$

Here, $\left(\alpha_{SAD}+\alpha_{hist}+\alpha_{height}=1\right)$ are the weights of each cost function, which are described next.

#### 3.2.2. Sum of Absolute Differences

In the bibliography, stixel matching is based on SAD applied pixel-wise over the RGB color scheme between frames $u_{i}\left\{t\right\}$ and $u_{j}\left\{t-1\right\}$. In [7], stixels are resized to measure 30 px. It is also used in the Results Section in order to compare the approaches.

#### 3.2.3. Histogram Matching

Our method relies on histograms to match stixels. Stixel size varies due to object position changes between frames or due to noise in the stixel height detection. To normalize this effect, a histogram is computed for each stixel, and a Hellinger distance between frames is calculated [38].

(4) $$\begin{array}{c} f_{hist}\left(u_{i}\left\{t\right\},u_{j}\left\{t-1\right\}\right)=2\times \sqrt{1-\underset{i=1}{\sum^{d}}\sqrt{H\left(u_{i}\left\{t\right\}\right)\left[i\right]\times H\left(u_{j}\left\{t-1\right\}\right)\left[i\right]}} \end{array}$$

H(u)[i] is the *i*-th bin in the histogram computed for stixel *u*, and *d* is the number of bins in the histogram. In our implementation, d=64, and the histograms are normalized.

Using this method to match stixels could lead to a bad score in certain circumstances, like in the extreme case in which both stixels have a constant, but almost similar brightness. In the unlikely circumstance that this happens, neighbor stixels will be properly matched. This will allow, in the next step, to correct these situations and match the stixels at the object level properly. This fact will be made clearer in Section 3.3.

#### 3.2.4. Height Difference

This metric is used to complement others, since by itself, it is not discriminative enough for a proper match, but it can help in the case of very similar scores in two or more possible matches. $f_{height}$ is computed as in Equation (5).

(5) $$f_{height}\left(u_{i}\left\{t\right\},u_{j}\left\{t-1\right\}\right)=1-\left|h\left(u_{i}\left\{t\right\}\right)-h\left(u_{j}\left\{t-1\right\}\right)\right|$$

h(u) is the height in real-world coordinates of the stixel in column *u*.

**Figure 4:**
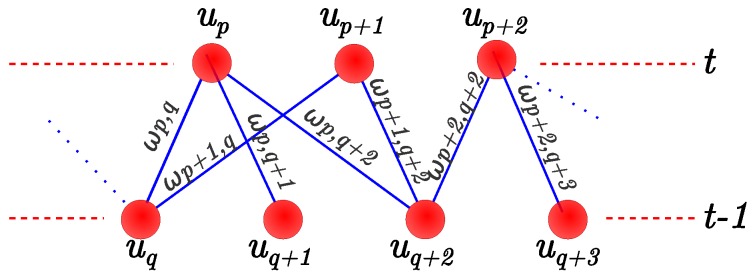
Bipartite matching graph representation for matching stixels between frames.

Section 4 shows the results for different $\alpha_{SAD}$, $\alpha_{hist}$ and $\alpha_{height}$. $f_{cost}$ is used to weight links between bipartite graph nodes. Figure 4 shows a representation of this graph. Nodes (stixels) at the current time are represented as $u_{p_{i}}$ and previous stixels as $u_{q_{i}}$. Match costs are assigned to edges as $\omega_{i,j}$. The minimization problem is shown in Equation (6).

(6) $$\hat{M}=\underset{M}{\arg\min}\sum_{(i,j)\in M}\omega_{i,j},\;\;\;\;\exists !\left(i,\cdot \right)\wedge \exists !\left(\cdot ,j\right)$$

A O(n·m·log(n)) Edmond’s maximum weighted matching algorithm [39] is used instead of DP [7]. This achieves better times and ensures that each match is performed one-to-one. In [7], a stixel can be matched with more than one stixel in the next frame. This complicates trajectory tracking, since multiple paths can be obtained for the same stixel. In our implementation, a matching set that maximizes the whole matching cost was chosen, ensuring that each stixel is matched with just one stixel.

### 3.3. Obstacle-Level Tracking

In this section, we describe the obstacle-level tracking. The first step is clustering, which joins every stixel with a similar depth into the same obstacle. The aggregation step fuses obstacles obtained from clustering with similar characteristics. In obstacle filtering, false obstacles are removed using obstacle motion and two-camera information. After these steps, obstacles are tracked between two consecutive frames. The algorithm steps are detailed in the following sections.

#### 3.3.1. Clustering

The first step is clustering, the goal of which is to join stixels with similar depths into fewer obstacles. Each obstacle consists of a set of similar stixels, from left to right. The Algorithm 1 is used for this step.
**Algorithm 1** Clustering algorithm.1:**function** CLUSTERING(Q{t})2:    O←∅3:    o←∅4:    **for** **each** stixel $q_{i}\in Q$, from left to right **do**5:        **if** $|depth\left(q_{i}\right)-depth\left(q_{i-1}\right)|>\tau_{depth\_dist}$ **then**6:           **if** $width\left(o\right)>\tau_{min\_width}$ **then**7:               o←∅8:           **end** **if**9:        **end** **if**10:        $o\leftarrow o\cup q_{i}$11:    **end** **for**12:**end** **function**

**Figure 5:**
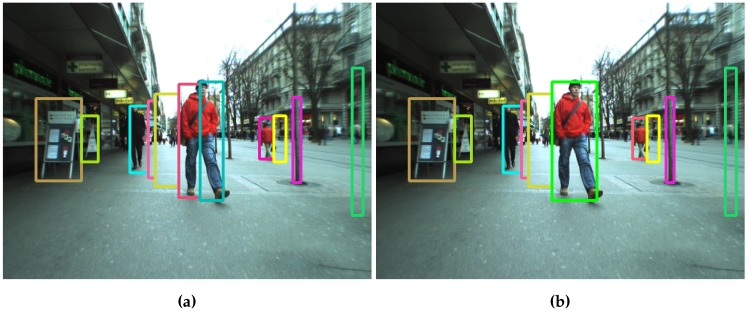
Obstacles detected before and after aggregation. The algorithm joins the stixels that belong to the same obstacles. (**a**) Obstacles before aggregation; (**b**) Obstacles after aggregation.

Q{t} are the stixels in current frame *t*. From left to right, stixels are accumulated until the depth difference between stixels is greater than $\tau_{depth\_dist}$. When the right border of an obstacle is reached, it is added to O, and the clustering process starts for new obstacles. If an obstacle is not wide enough, it will be rejected. Stixels generated due to noise, as shown in Figure 1, are removed. O also includes parameters, such as object depth, which is computed as the minimum depth between all of the clustered stixels. Figure 5a shows the results after clustering.

##### Obstacle Aggregation

Sometimes, stixels are located at a depth different from their real position, as shown in Figure 5a where the legs of a person in the foreground are separated enough to show the ground between them. This confuses the detection process, which regards the obstacle’s base as the central part of this person and not his feet. The process described in Algorithm 2 reduces this effect.
**Algorithm 2** Aggregation algorithm.1:**function** AGGREGATION(O)2:    $O^{\prime }\leftarrow \emptyset$3:    $o^{\prime }\leftarrow \emptyset$4:    **for** **each** object $o_{i}\in O$, from left to right **do**5:        **if** $|X\left(o_{i}\right)-X\left(o_{i-1}\right)|>\tau_{lateral\_aggregation\_dist}$ **or** $|Z\left(o_{i}\right)-Z\left(o_{i-1}\right)|>\tau_{depth\_dist}$ **then**6:           $O\leftarrow O\cup o^{\prime }$7:           $o^{\prime }\leftarrow \emptyset$8:        **end** **if**9:        $o^{\prime }\leftarrow o^{\prime }\cup o$10:    **end** **for**11:**end** **function**


All previously-detected obstacles are tested, again from left to right. If the lateral distance (in world coordinates) is less than $\tau_{lateral\_aggregation\_dist}$, the depth difference is checked again. If it is less than $\tau_{depth\_dist}$, the two obstacles are joined. Figure 5 shows this process. In the left image, the person in first plane is divided into two different obstacles. After aggregation, this is assigned to a single obstacle. The final obstacle depth between the two obstacles is regarded as minimal.

##### Obstacle Filtering

Figure 5b shows some false obstacles, such as those between the two pedestrians on the left side of the image (in pink and yellow). There is another next to the man in the background (yellow) and the last one on the right side of the image (green). Signs and poles are not considered false obstacles, since they are elements to be avoided.

In order to distinguish real obstacles from false ones, the images captured are recorded so that motion can be detected. Motion can originate both from obstacles (i.e., a person walking) and camera movement. This allows detecting occluded or changing areas so that new obstacle borders can be detected.

**Figure 6:**
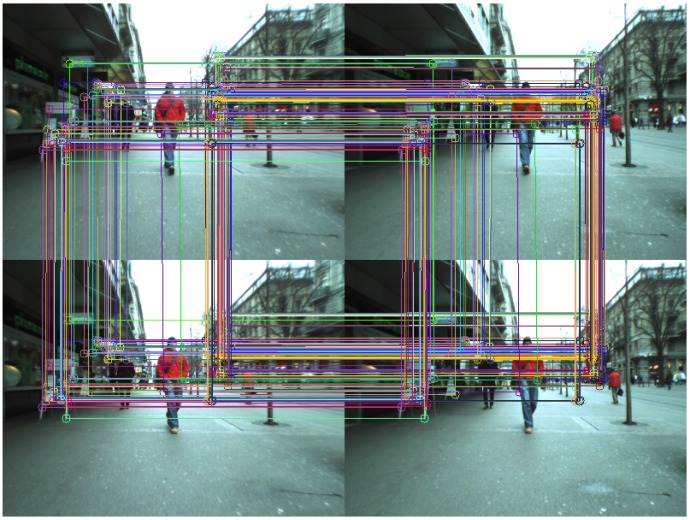
Common points between frames *t* and t−1 in the matching cycle.

The search for correspondences between the two images relies on polar rectification, as in [40]. The first step defines the common region between images, so the epipoles and the homography *H* must be calculated using the fundamental matrix *F* [41]. Epipolar geometry is described by Equation (7).

(7) $$m_{L,t-1}^{T}\times F\times m_{L,t}=0$$

where $m_{L,t-1}$ and $m_{L,t}$ are homogeneous representations of corresponding image points in the left image of frames *t* and t−1. Correct correspondences must be obtained in order to yield *F*, so they are computed in the following order [17]: $I_{L,t}\to I_{R,t}\to I_{R,t-1}\to I_{L,t-1}\to I_{L,t}$, where $I_{\{L,R\},t}$ is the left (*L*) or right (*R*) image in frame *t*. From an initial set of features in $I_{L,t}$, valid matches in $I_{R,t}$ are obtained. The cycle is complete when $I_{L,t}$ is reached, keeping only valid matches. Figure 6 shows the results of the matching process, where each matching cycle is represented by the same random color. A match is valid if the following holds:

- The points obtained should be the same for the entire cycle.
- Features in $I_{L,t}$ must be in the same row as $I_{R,t}$. The same applies to $I_{L,t-1}$ and $I_{R,t-1}$.
- The distances between features in frames *t* and t−1 should be similar.

**Figure 7:**
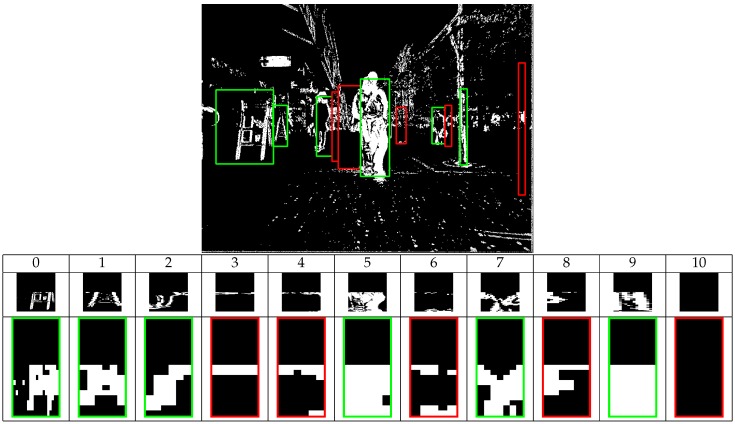
Object filtering phase. Binarized motion image (**top**) (k=0.2s); Occupancy maps on the ground (**bottom**).

In order to detect changed pixels and to remove false stixels, frame *t* is aligned to t−k [40] to obtain a pixel-wise absolute difference. A stixel is considered valid if it is consistent in the left and right images, in the current and previous frame. Figure 7 shows this difference thresholded, binarized and projected back to current image coordinates. Small noise differences are rejected. For each obstacle, its Region Of Interest (ROI) is determined, and its top half is rejected, meaning the algorithm only looks for obstacle motion close to ground, since obstacles usually exhibit more motion in their lower half (legs or wheel movements). In static obstacles, the motion due to camera movement is more or less uniform throughout the entire object. Changes due to perspective are also small over planar ground.

The points obtained after the thresholding process are located in their corresponding position in 3D coordinates. The ground is divided into an occupancy grid of 10×10 cm cells. When a point falls in the cell, it is marked as occupied. Figure 7 shows examples of motion, ROI and an occupancy grid. Real obstacles, like 5 or 7, exhibit higher densities compared to 4. Even the motion of 2 (a man in a black suit where the colors complicate detection) is properly detected. To improve detection, a frame is not compared to the one immediately preceding it, but to that corresponding to t−k (in seconds, where *t* is the current time), which makes differences due to motion more noticeable. In our tests, k=0.2 s, which, despite being a conservative value, makes the differences appreciable. Obstacles are rejected as per Equation (8).

(8) $$false\left(o\right)=\begin{array}{c} \left\{\begin{array}{cc} true & \text{if}\;\frac{count(G_{o},true)}{count\left(G_{o},true\right)+count\left(G_{o},false\right)}>\tau_{occ}\\ false & otherwise \end{array}\right. \end{array}$$

$count(G_{o},j)$ counts occupied cells in the occupancy grid $G_{o}$. $\tau_{occ}$ is the threshold parameter. The width of each obstacle in real-world coordinates is also checked. Figure 7 shows rejected (red) and accepted (green) obstacles.

#### 3.3.2. Tracking

The first tracking method is based on Section 3.2.1, where the initial stixel level matching is used to maximize matches between obstacles. The second one matches directly using template matching techniques since the differences between frames are small. The results from applying both methods are shown in Section 4.3. The first method exhibits better recall along frames; however, the second is faster, with lower, but acceptable, recall.

##### Two-Level Tracking Approach

The tracking problem is regarded as a pair matching process repeated over time. The correspondence matrix $C_{\left|O\{t\}|\times |O\{t-1\}\right|}$ stores the number of correspondences between stixels in current and previous frames. The tracking process is described in Algorithm 3.
**Algorithm 3** Two-level tracking algorithm.1:**function** TRACKING(O{t}, O{t−1})2:    $C_{\left|O\{t\}|\times |O\{t-1\}\right|}\leftarrow 0$3:    **for** **each** object o{t}∈O{t} **do**4:        **for** **each** stixel q{t}∈o **do**5:           Find correspondence q{t−1} for q{t}6:           Find the object o{t−1}∈O{t−1} associated to q{t−1}7:           **if** o{t−1} found **and** $∥o\left\{t\right\}-o\left\{t-1\right\}∥<\tau_{max\_obst\_dist}$ **then**8:               C(o{t},o{t−1})←C(o{t},o{t−1})+19:           **end** **if**10:        **end** **for**11:    **end** **for**12:**end** **function**


**Figure 8:**
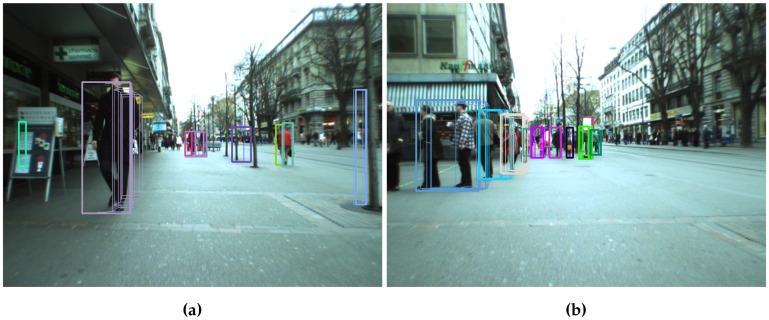
Two-level based tracking algorithm results. In the image, the stixels detected in the current and previous frames are superimposed. Both frames were extracted from Bahnhof sequence. (**a**) Frame 30; (**b**) Frame 320.

Two objects are associated between frames if there is at least one stixel correspondence and they are sufficiently close, assuming that the motion between frames is small (if the frame rate is high). Matched pairs $\hat{C}$ are obtained by solving the maximization problem in Equation (9) using a correspondence matrix. Each track is stored in an internal structure that associates tracks with obstacles, allowing for the inclusion of new obstacles. The results are shown in Figure 8, Section 4 and in the method pipeline video.

(9) $$\hat{C}=\underset{C}{\arg\max}\sum_{(i,j)\in M}C\left(i,j\right),\;\;\;\;\exists !\left(i,\cdot \right)\wedge \exists !\left(\cdot ,j\right)$$

##### Object Tracking Approach

**Figure 9:**
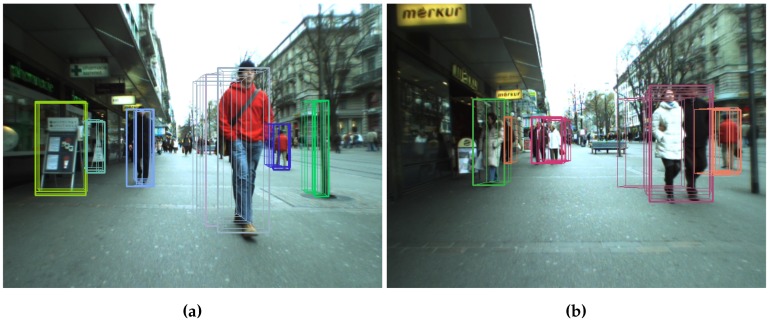
Object-based tracking results. In the image, the stixels detected in the current and previous frames are superimposed. Both frames were extracted from Bahnhof sequence. (**a**) Frame 15; (**b**) Frame 126.

A cost matrix (Equation (10)) is not generated using stixels associated with obstacles, since this information is not available. The histogram difference described in Section 3.2.1 is used, but for each pair of obstacles and not at the stixel level. The tracking problem thus becomes the same as in the two-level tracking case, in which Equation (9) is maximized. Figure 9 and Section 4 show some tracking results.

(10) $$\begin{array}{cc} & C(o\{t\},o\{t-1\})=\\ & \;\;\;\;\;\;1-\left(2\times \sqrt{1-\underset{i=1}{\sum^{d}}\sqrt{H(o\{t\})[i]\times H(o\{t-1\})[i]}}\right) \end{array}$$

#### 3.3.3. Integration with the Navigation Subsystem

This work is intended to provide the input for the navigation subsystem of our autonomous vehicle, Verdino. The navigation scheme is an adaptation of [42] using [6] as the localization system. It computes a set of tentative trajectories based on the Frenét space [43,44] (which is shaped according to a global plan, which connects the current position to a given target [45,46]). These trajectories are projected back to Euclidean space. Tentative paths are weighted, using factors such as length, curvature and safety. A layered costmap [47] is used to connect the navigation subsystem and obstacle detection using an occupancy grid. Information on obstacles (stixels and their motion) is stored or updated by marking them on the map. The costmap consists of two different layers.

The first layer represents the stixels in the current frame, projected and transformed to map coordinates. The technique of growing the obstacles allows planning the vehicle’s movements as if it were a point, without occupying space, which simplifies the planning. Every obstacle detected by the vision module is grown to vehicle size, so that the vehicle will not crash into obstacles even when the vehicle’s planning does not consider size (Layer 1). The world map is a grid in which obstacles are represented using values from 0 to 255, where 0 represents a free area and 255 an obstacle. The cost of each cell c(x,y) in the map is calculated using Equation (11). This cost is used by the autonomous vehicle to calculate a safe path that avoids the obstacles detected by this stixel method.

(11) $$cost\left(c\right)=253\times e^{\beta \times (\rho -∥nearest(c)-c∥)}$$

β is a scaling factor that defines the cost function’s slope; nearest(c) is the nearest obstacle cell; *c* is the current position; and ρ is the circumscribed radius of the vehicle.

**Figure 10:**
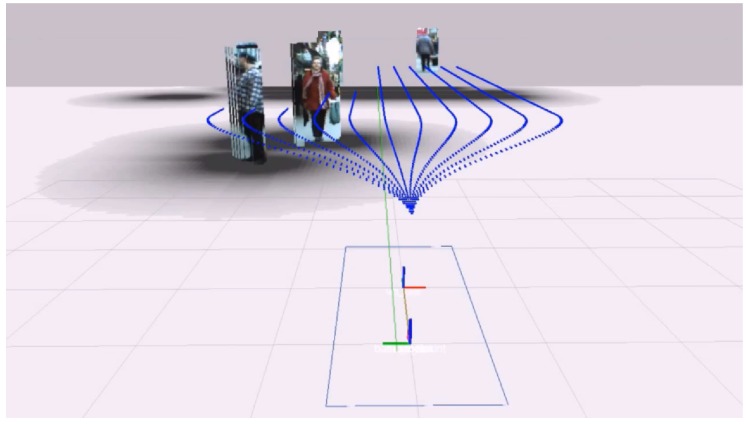
Navigation subsystem integration with stixel detection. A gray-scale costmap layer is included where black represents an obstacle and white is free space. The gray scale is generated using Equation (11). The possible routes that the vehicle can take are shown in blue.

The second layer represents the obstacle’s motion, transformed and referenced to the map. A Kalman filter is applied to past trajectories to predict future ones. Obstacle growth is also carried out in this layer, but the vehicle is allowed to approach the possible future positions of obstacles more than it is allowed to approach them in the current position (Layer 1). Figure 10 shows the navigation subsystem integration. Tentative trajectories are long in free areas and short when close to obstacles. The attached video stixel world-based navigation shows a full navigation sequence.

## 4. Results

Four factors were considered when evaluating the method:

1. The quality of the clustering process.
2. Stixel depth accuracy compared to object-level tracking.
3. How well tracks are recalled under various conditions.
4. Computational time.

The results obtained in this paper are compared to [7,37] using the Bahnhof sequence [26] (7400 obstacle annotations, height ≥40 px, 999 stereo pairs, 640×480 pixels, 15 fps).

### 4.1. Clustering

**Figure 11:**
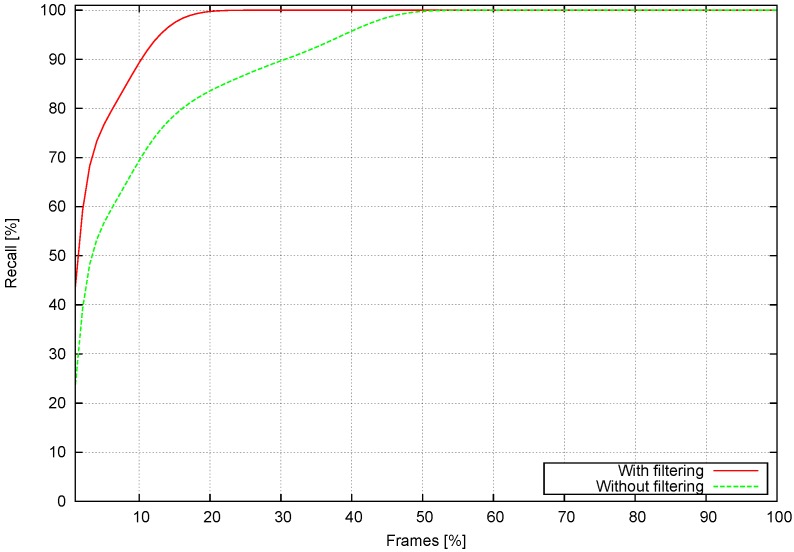
Obstacle detection rate as a function of the number of frames analyzed for a sequence.

This test is applied to the clustering method described in Section 3.3.1. Detections are compared to actual obstacles in each frame. The method is tested with and without filtering, as described in Section 3.3.1. Figure 11 shows its results.

Figure 11 shows, ordered by recall, the whole sequence processed frame by frame. The total sequence frame percentage is on the *x* axis and the recall on the *y* axis. The results of the stixel detection method are compared to the annotations included in the dataset in order to calculate recall. The graph indicates that analyzing only one frame yields a recall rate of 50% for the filtered option and 30% for the non-filtered option for all of the obstacles included in the whole sequence, which are presented in the current frame. If only 10% of the sequence is analyzed, the recall grows to 90% in obstacle detection (70% error in the non-filtered option). This means that by analyzing just 10% of the frames in the sequence, 90% of the obstacles present in those frames can be detected. Analyzing 20% of the frames in the sequence yields a 100% recall rate (55% of frames in the non-filtered version).

**Figure 12:**
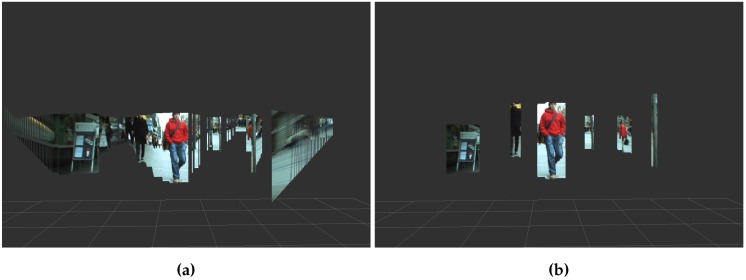
Stixel comparison between [2] and this paper in the same frame. (**a**) Stixels detected by [2]; (**b**) Stixels detected by our method.

Figure 12a, shows the original stixels (projected in 3D) with considerable noise (especially between obstacles) and free areas detected as obstacles. In Figure 12b, only obstacle stixels are represented, with the depths restored after the clustering process.

### 4.2. Stixel Accuracy

**Figure 13:**
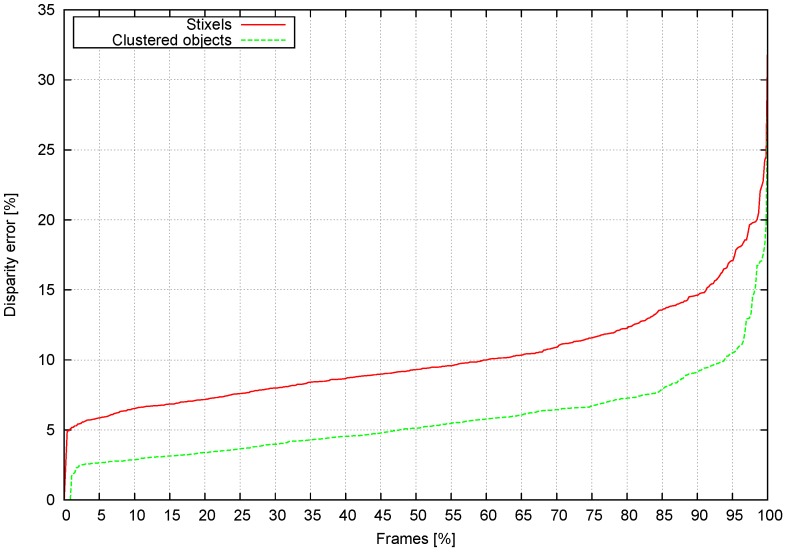
Disparity difference between stixel and object clustering.

Stixel depth after clustering is compared to the disparity map shown in Figure 13 and used as the ground truth. The error in the pixels is calculated as an average of disparity differences between the stixel depth and the disparity map using Equation (12).

(12) $$error=\frac{\sum_{q_{i}\in Q}\;\sum_{v\in V}∥disp_{GT}\left(v,q_{i}\right)-disp_{Q}\left(q_{i}\right)∥}{N\times d_{max}}\times 100$$

$V=\{b\left(q_{i}\right),\ldots ,t\left(q_{i}\right)\}$, $b(q_{i})$ is the bottom row of stixel $q_{i}$; $t(q_{i})$ is the top row of stixel $q_{i}$; $disp_{GT}\left(i,j\right)$ is the ground truth disparity at a certain row *i* and column *j*; $disp_{Q}\left(q_{i}\right)$ is the disparity computed for the stixel $q_{i}$; *N* is the total number of pixels being compared; and $d_{max}$ is the maximum disparity allowed.

**Figure 14:**
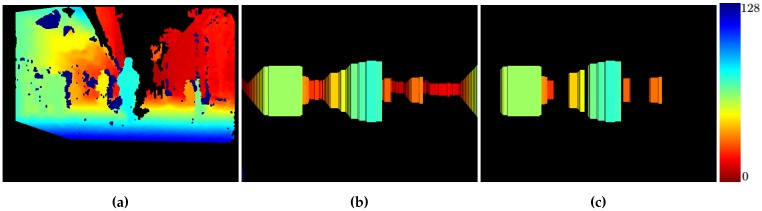
Comparison between the ground truth, stixels and reconstructed objects. The color code represents distance to the camera. (**a**) Ground truth; (**b**) Obtained stixels; (**c**) Reconstructed objects.

Figure 13 shows the error for each frame in a sequence in ascending order. The stixel error (red) grows faster than the clustered obstacle error (green). Approximately 95% of the frames with clustered obstacles have a disparity error below 10%. However, just 60% of the frames exhibit a disparity error below this value with the original stixel computation. Figure 14 shows the ground truth disparity map, the original stixels [37] and the clustered obstacles in a color scale where red represents lower disparities (further) and blue higher disparities.

### 4.3. Tracking

In this section, tracking evaluation tests are shown in terms of the recall measured using two different criteria: tracking capabilities after a few frames (track length) and performance when the time between frames is increased. Table 1 shows a selection of the most representative configurations.

There are two configurations based on [7]: the first one just uses the SAD cost, and the second one is the final configuration described in [7]. Configurations 3 to 6 apply the method presented in Section 3.3.2 (two-level tracking approach), where Configuration 1 and 2 parameters are used, plus an evaluation of $\alpha_{hist}$ factor. Configuration 7 presents object-based tracking results (Section 3.3.2, object tracking approach).

#### 4.3.1. Sequence Performance

**Figure 15:**
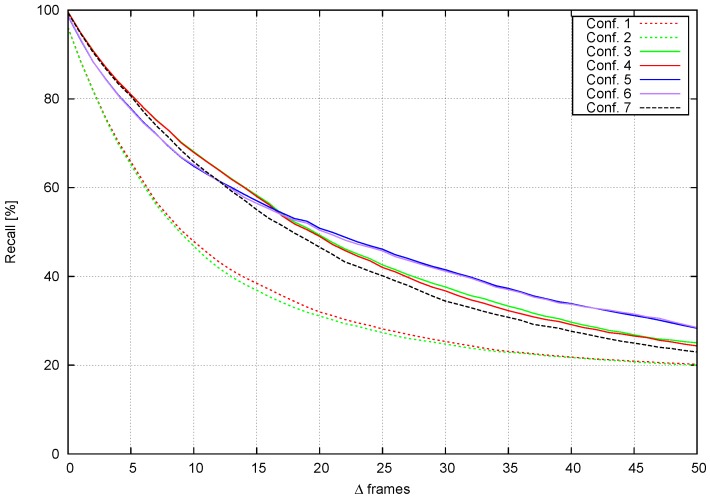
Recall obtained with different configurations.

Tracking capabilities are evaluated as per [7], using annotated obstacle bounding boxes as the ground truth. Each configuration evaluated predicts bounding box positions up to Δ frames in the future. For each frame, recall is evaluated using the intersection over the union metric. Figure 15 shows the recall vs. Δ frames evaluation starting from every frame in the video sequence.

**Figure 16:**
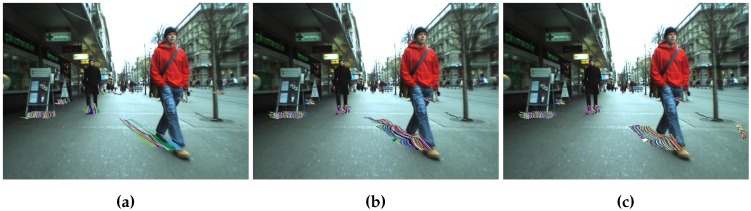
Stixel level tracking with Configurations 1, 5 and 7. (**a**) Configuration 1; (**b**) Configuration 5; (**c**) Configuration 7.

Configurations 1 and 2 [7] fall quite fast, with a recall below 70% after just five frames. Furthermore, the $\alpha_{height}$ contribution is not clear. Two-level tracking methods yield better results, especially when $\alpha_{hist}\neq 0$. The second tracking level filters much of the noise, making tracking more reliable. Figure 16 shows qualitative results for Configurations 1, 5 and 7. The trajectories obtained for Configuration 5 are the longest and smoothest, and the effect of avoiding multiple matches for the same stixel are also evident. In Configuration 1, the trajectories for many stixels start from the same single stixel. Configurations 5 and 6 use $\alpha_{hist}$ and Configurations 3 and 4 $\alpha_{SAD}$. Histograms are normalized just before matching, while the sum of absolute differences is done pixel by pixel. This results in longer tracks in Configurations 5 and 6, since matching is more robust to illumination changes.

Object-based tracking (Configuration 7) shows good results for the first few frames, but it falls faster than two-level-based methods, since two-level tracking is more tolerant to clustering errors. If in one frame, a relatively large portion of the background is considered an obstacle, the histogram will change, and the matching score could be small. Figure 16 shows comparable quality tracks in Configurations 5 and 7, but 5 achieves longer tracks.

#### 4.3.2. Performance at Different Frame Rates

**Figure 17:**
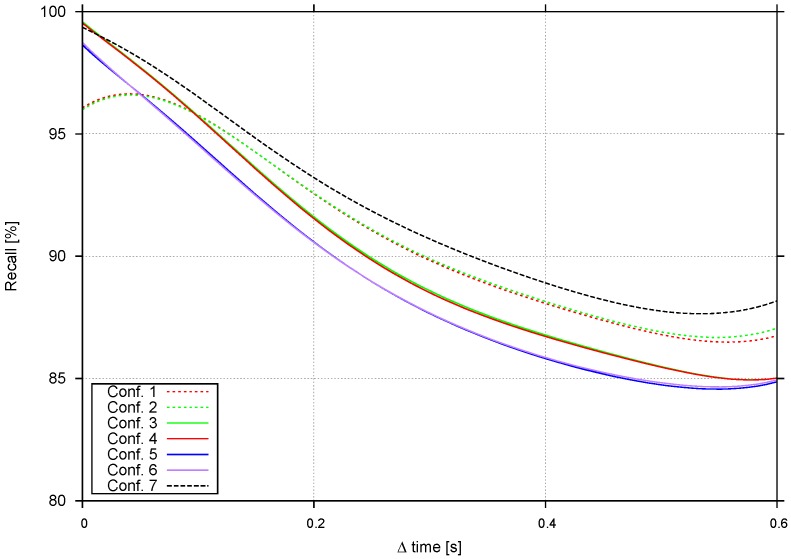
Recall of different configurations vs. frame rates.

In this section, we analyze recall as a function of Δ frames. The tests from the previous section are repeated, but now, the time step between frames is increased *k* frames each time, with k=0.06,⋯,1.2s (from 1 up to 20 fps, in a 15-fps video). Figure 17 shows recall versus time step increment. Four different profiles are detected involving Configurations 1 to 7. This tests also confirms that $\alpha_{height}$ is negligible. The most tolerant configuration is 7, since the object-level based tracking is able to handle slightly larger changes than stixel-level tracking.

**Figure 18:**
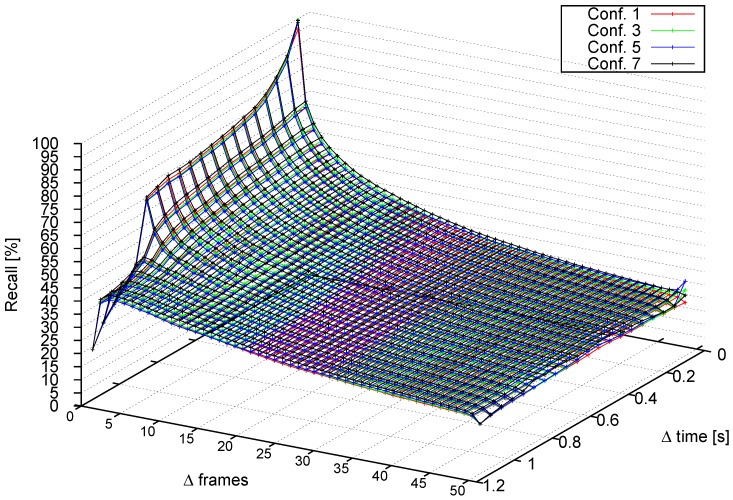
Tracking capabilities at different frame increments for Configurations 1, 5 and 7.

Figure 18 compares recall, Δ frames and Δ time for Configurations 1, 3, 5 and 7. When Δ frames ≈0, the pattern shown in Figure 17 is repeated. However, when Δ frames starts to increase, Configurations 3 and 5 do not fall as fast as Configuration 1, which confirms the conclusions drawn from previous tests. Configuration 7 achieves a higher recall than the other configurations.

#### 4.3.3. Performance with Other Sequences

In order to assess the performance of our algorithm in situations other than those found in the Bahnhof sequence, other sequences were processed, yielding the results described in this section. The sequences studied were Sunnyday, Jelmoli and Loewenplatz. The last one is quite interesting, since it was not obtained in a pedestrian area, the main focus of our application, meaning faster changes between frames. It will also allow us to ascertain how our algorithm behaves in an environment for which it was not originally designed.

The algorithm was also tested in our own sequences, called Herradores, Carrera and Trinidad, which were taken in the areas in which the vehicle is expected to operate. Since there is no ground truth available for those sequences, only some examples of the output obtained are shown in this section.

**Figure 19:**
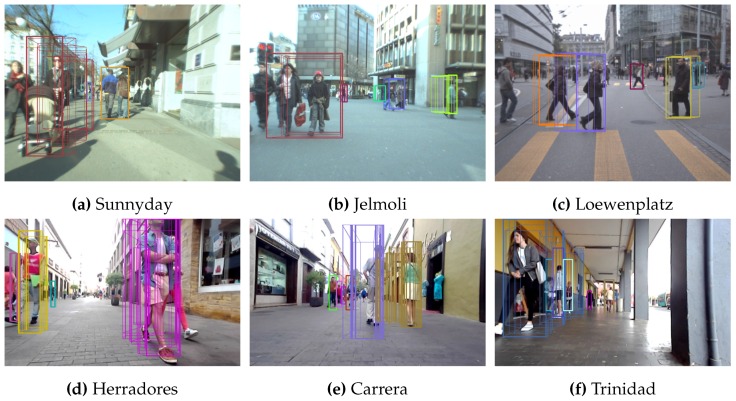
Other sequences processed. The top row shows the output for three very well-known sequences. The bottom row shows the results for the sequences obtained by our vehicle, Verdino, in the environment in which it will operate. (**a**) Sequence Sunnyday from ETHZ dataset; (**b**) Sequence Jelmoli from ETHZ dataset; (**c**) Sequence Loewenplatz from ETHZ dataset; (**d**) Sequence Herradores obtained from prototype Verdino; (**e**) Sequence Carrera obtained from prototype Verdino; (**f**) Sequence Trinidad obtained from prototype Verdino.

In Figure 19, we can see that the algorithm is able to detect the pedestrians and follow them along their paths. Sequences Herradores and Carrera are quite challenging since the horizontal lines in the cobblestone can confuse the algorithm, but it was able to handle this with no apparent problems.

**Figure 20:**
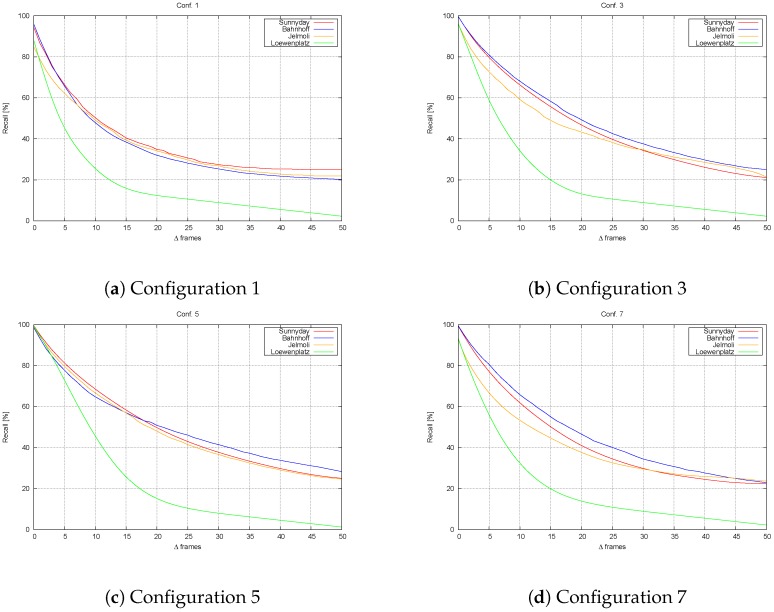
Recall obtained for the sequences tested. Note that our algorithm outperforms that in [7] for every sequence, especially Configuration 5. (**a**) Recall obtained with Configuration 1; (**b**) Recall obtained with Configuration 3; (**c**) Recall obtained with Configuration 5; (**d**) Recall obtained with Configuration 7.

Figure 20 shows a comparison of the output obtained for those sequences for which a ground truth was available for Configurations 1, 3, 5 and 7. Configurations 2, 4 and 6 are not shown, both for clarity reasons and because, as previously shown, the differences between them and Configurations 1, 3 and 5 (respectively) are negligible.

Again, our method offers a clear improvement over the one presented in [7]. Furthermore, in Configurations 3 and 5, there is a noticeable improvement associated with our use of histogram comparisons for measurement. Note as well that in pedestrian environments, the behavior is similar. However, the use of the algorithm in vehicles exhibits worse behavior, since, as shown by the Loewenplatz sequence, the performance is significantly reduced. The main reason for this is the large changes in the images due to an increase in the vehicle’s speed. This is confirmed by the results obtained for Configuration 7, the results of which are not as degraded as they were for the other configurations, for that sequence.

### 4.4. Computation Time

**Figure 21:**
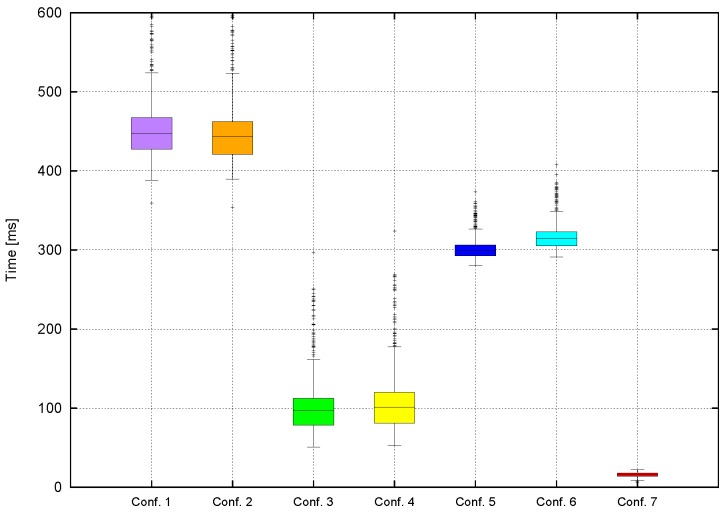
Times obtained for each configuration.

Figure 21 shows that the fastest configuration is 7. This is to be expected, since only object comparisons are involved and few obstacles are compared in each frame, vs. the 640×640 comparisons for the worst case in the stixel-level tracking. Figure 21 also shows that graph-based methods are slightly faster.

The algorithm was tested using Verdino’s onboard computer, an i7-3770K processor with 16 Gb of RAM DDR-3 memory, SSD storage and an NVIDIA GeForce GT 640. Every method was implemented modularly using an Indigo ROS [48] Ubuntu-based distribution. The navigation method is able to work in real time for an autonomous vehicle, the implementation of the method is available at [49].

## 5. Conclusions

In this paper, we present an innovative object tracking method based on the stixel world [1] and applied to driver assistance. Our work expands and improves upon that presented in [7]. The use of a two-level tracking system offers robust stixel tracking, and the obstacle-based approach provides robustness, even at low frame rates. Once the output of the method is connected to the navigation subsystem through a layered costmap, it is ready to be used in our platform, Verdino, or in an autonomous car.

A simple, but effective clustering method based on stixels is introduced that yields a good detection rate. Moreover, we have shown how these clustered objects can be used as the basis for reconstructing the initial disparities, offering noticeable improvements and reducing the disparity error by almost one-half.

The results obtained by several configurations were evaluated. Two of them correspond to [7]; four of them present different parameter configurations for the two-level based approach; and a last configuration is based on the obstacle tracking approach.

The performance obtained along the sequence was measured in terms of recall, with the two-level-based method exhibiting better results than the others. The most important factor in the algorithm is $\alpha_{hist}$, followed by $\alpha_{SAD}$. The contribution from $\alpha_{height}$ is negligible. The obstacle-based approach does not seem to be a good choice when the frame rate is high, but it offers a good solution at lower frame rates, since it is more tolerant to large changes between images. It is also the fastest, making it a good choice to save computational resources.

The method works in real time using both variants, and it is fully integrated into Verdino, providing a fast, vision-based reconstruction of the environment. The method is ready to be used for navigation purposes in obstacle avoidance tasks. Videos demonstrating the effectiveness of the method in dense environments are also included.

## Figures and Tables

**Table 1:** Parameter configurations results.

	$\alpha_{\mathrm{SAD}}$	$\alpha_{\mathrm{hist}}$	$\alpha_{\mathrm{height}}$	
Configuration 1	1	-	0	Gunyel et al. [7]
Configuration 2	0.5	-	0.5	Gunyel et al. [7]
Configuration 3	1	0	0	Two-level tracking
Configuration 4	0.5	0	0.5	Two-level tracking
Configuration 5	0	1	0	Two-level tracking
Configuration 6	0	0.5	0.5	Two-level tracking
Configuration 7	-	-	-	Object tracking

## Supplementary Materials

The following are available online at http://www.mdpi.com/1424-8220/16/8/1182/s1: Video S1: MethodPipeline.mp4. Video S2: stixelsNavigation.mp4.

## Abbreviations

The following abbreviations are used in this manuscript:

ADAS: Advanced Driver Assistance Systems
LIDAR: Light-Detection And Ranging
ROI: Region Of Interest
